# Breaking Barriers: The History of Women's Education and the Training of Female Surgeons

**DOI:** 10.1111/ans.70686

**Published:** 2026-04-18

**Authors:** John P. Collins

**Affiliations:** ^1^ Department of Surgery University of Auckland Auckland New Zealand

## Abstract

**Background and Aims:**

Medicine and surgery have been practised by women since the earliest of times, but as these activities became professionalised, they became excluded by various barriers. The aims of this review are to identify these obstacles and how they were overcome.

**Methods:**

An analysis was undertaken of the history of women's education and of female surgeons, and of the contemporary factors which influenced their development.

**Results:**

Victorian assumptions about gender differences and societal roles resulted in a lack of educational opportunities for girls and women, their exclusion from university and medical college examinations, and from opportunities to train in hospitals. Claims that the anatomy dissection room and the operating theatre were unsuitable environments for women were further obstacles for those who wished to become surgeons. Examination of the lives of those women who led the changes in education, and of the first females to obtain the Fellowship of the different Royal Colleges of Surgeons by examination, or to become Board Certified in the US, illustrates the challenges they overcame and the important roles they played in health care and medical research. Despite their success and that of the countless female surgeons who have since followed them, challenges remain.

**Conclusion:**

Analysis of the history of the education of young women and of the first female surgeons demonstrates the barriers and unfounded biases they overcame. To achieve the satisfactory recruitment, education, training, career success and retention of women surgeons, lessons must be learned from history and the remaining obstacles overcome.

## 1 Introduction

The history of women's pursuit of medical and surgical education was marked in 2025 by several important anniversaries. It was the 170th anniversary of the graduation from medical school of the first woman to practice as a surgeon in the U.S. Army, and the 160th anniversary of the first woman to qualify as a medical practitioner in the United Kingdom (UK). It was also the 130th anniversary of first woman to hold a master's degree in surgery in the UK which enabled her to be appointed as the first female consultant surgeon. These important milestones provided a timely opportunity to review and reflect on the evolution of the education and training of women surgeons. A crucial part of this story is how the barriers to the education of girls and women were overcome before those who wished to be surgeons could realise their dreams.

## 2 Methods

A review was undertaken of historical sources relating to the education of girls and women and of the first female medical practitioners and surgeons, between the end of the eighteenth century and the middle of the twentieth. This included an examination of the contemporary social, cultural, religious, and political contexts within which these developments took place.

## 3 Findings

In his reply to a toast to the guests at a luncheon given in 1944 by The Royal College of Physicians of London, the British Prime Minister Winston Churchill remarked regarding the history of medicine that, ‘The longer you look back, the farther you can look forward’ [1]. The earliest formal obstruction to women becoming surgeons was included in the Charter given by Henry VIII to the Barber Surgeons in 1540, through the declaration that ‘no carpenter, smith, weaver or women shall practice surgery’. However, the likelihood of women becoming surgeons over the following 250 years was extremely remote due to the prevailing attitudes to women's place in society. Women were expected to marry and perform household and motherly duties rather than to seek formal education.

The Industrial Revolution and urbanization of the population during the late eighteenth and early nineteenth centuries, resulted in major socio‐economic and cultural changes in society. A new and increasingly influential middle‐class emerged leading to a rapid expansion in the demand for education, which brought the inadequate schooling for girls and women to the fore. A new type of manners writing also appeared in the etiquette books which replaced the previous courtesy and conduct literature [2]. Rather than focusing on attracting a husband through their domestic abilities as occurred previously, girls were encouraged to learn a list of skills known as ‘accomplishments’ [3]. These are summarised in chapter 8 of Jane Austen's novel of manners and romance *Pride & Prejudice* first published in 1813 [4].

Those individuals who began to challenge the lack of a proper education for girls and women were impeded by the class divisions in the social structure of society, the rigid assumptions about gender differences and roles, and the barriers by reason of religion and colour. The Victorian vocabulary of class and gender dominated assumptions about a person's place in society and the function of the different social institutions. Education did not escape this thinking, and even when Victorians began to acknowledge the need for education, they maintained ‘there should be different kinds and levels of schools with different objectives for each social class and gender’ [5].

Victorian assumptions about women and their place in society held they were not suited by temperament or intellect for the clergy or public life, and not capable of the sustained rigorous work of university studies. Furthermore, many contemporary men and women alike believed that advanced education would spoil women's ‘cherished innocence and nurturing instincts’ [6]. Moreover, men and women were regarded as different not only biologically, but also in their intellect, psychology, and emotions. These philosophies were used to support a belief in separate spheres—the ‘public’ for men and the ‘private’ for women [5].

Added to these challenges was the Victorian philosophy of ‘Manliness’ which replaced the gentle Georgian sensibility of the late Romantic period. Loyalty, bodily vigour, mental toughness and emotional restraint, physical courage, endurance and discipline became the dominant philosophy. This newly held belief of ‘male manliness’ versus the female ‘angel in the house’ [7] permeated society. Dr. John William Burgon—later Dean of Chichester Cathedral—and a strong opponent to the admission of women to Oxford University declared in an allocution to women during his sermon in New College Chapel Oxford in 1884 that, ‘Inferior to us God made you, and inferior to the end of time you will remain’ [8]. It is said the women present threw their kneelers at this bachelor after he had finished speaking!

Overcoming the obstacles to women's education and their overall progress in society took a gathering storm to sweep them aside. This was led by women reformers and their male allies, social movements, cultural shifts and law reforms. The first was the writer and women's activist Mary Wollstonecraft, who wrote in 1792, ‘Let woman share the rights and she will emulate the virtues of man; for she must grow perfect when emancipated’ [9]. Six years later the acclaimed actress, poet, dramatist and novelist Mary Robinson published a pamphlet under a pseudonym, containing a critique of female disempowerment, and campaigned for their access to the traditionally male ‘liberal education’, and argued for a ‘system of mental equality’ [10].

In 1866, the English feminist and suffragist Emily Davies published a groundbreaking work advocating for women's university education and their empowerment [11]. This was followed in 1869 by an essay published by the influential English philosopher, political economist and politician John Stuart Mill. Mill argued that the existing ‘legal subordination of one sex to the other – is wrong in itself, and now one of the chief hindrances to human improvement; and that it ought to be replaced by the principle of perfect equality, admitting no power or privilege on one side, nor disability on the other’ [12]. While these well‐researched and reasoned arguments for equality between the sexes were an affront to the existing conventional norms, their far‐reaching implications could not be ignored.

Although a handful of schools for girls and women began to be established in Europe and the US by the late eighteenth century, it was not until the mid‐nineteenth that this occurred to any great extent in Britain. The first was Queen's College, London, founded in 1858 to provide ‘a serious education for young women’. One year later the liberal and non‐sectarian Bedford College for Women was established. Cheltenham Ladies' College followed in 1853 and was led for 50 years by the influential educational reformer and suffragist Dorothy Beale. Other girl's schools and women's colleges were gradually founded elsewhere.

As a result, an increasing cohort of young women became accomplished and more learned, but the battle for the recognition and acceptance of their achievements and their access to university education had yet to be won. Well educated and accomplished girls were considered a threat to the socially accepted rules for behaviour of women within the Victorian society and advised to ‘soften their erudition with a graceful and feminine manner’ [3]. This was to avoid being called a ‘blue stocking’, the name given to those who had devoted themselves too enthusiastically to intellectual pursuits rather than traditional roles. They were even considered to be ‘unfeminine and off‐putting in the way they attempted to usurp men's ‘natural’ intellectual superiority’, and possibly even unmarriageable [3].

## 4 Campaign for University Education for Women

Access to university education for women commenced in Great Britain with their admission to the University of London programmes in 1867. However, despite their success in passing the university exit examination in 1869, they were awarded Certificates of Proficiency rather than the degrees which were awarded to male students. The experiences of these first women known as the ‘London Nine’ [13], and of the ‘Edinburgh Seven’ medical students rejected by the University of Edinburgh—notwithstanding their continuing academic success [14], gained national attention and support. Their campaign led by the formidable Sophia‐Jex‐Blake (one of the Edinburgh seven) [15] and others placed demands for university education for women on the national political agenda.

The Medical Act 1876 repealed the previous Medical Act of 1858 and ruled that all qualified applicants, whatever their gender, must be allowed admission to the examinations necessary to qualify as medical practitioners. The University of London opened full degrees to women in 1878, thus becoming the first British university to do so. Other universities soon followed except for Oxford and Cambridge, where degrees for women were not granted until 1920 and 1947 respectively [16].

## 5 The Making of Medical Women and Surgeons

It is therefore not surprising that those women who wished to access medical education in the mid to late nineteenth century faced many barriers [17, 18]. The first woman to overcome these challenges was the British‐born Dr. Elizabeth Blackwell (1821–1910). After many rejections she eventually won a place in Geneva Medical College New York in 1847. Despite initial discrimination from the male student body and the local townspeople, she graduated first in her class and with a gold medal in 1849. Blackwell wished to train as a surgeon but was unable to secure the necessary hospital positions in the US or Britain. She moved to Paris where she became the first woman to study surgery. However, the loss of sight in one eye due to contracting purulent ophthalmia infection precluded her wish to be ‘the first lady surgeon in the world’ [19]. She became the first woman to be registered on the British Medical Register in 1856.

**FIGURE 1:**
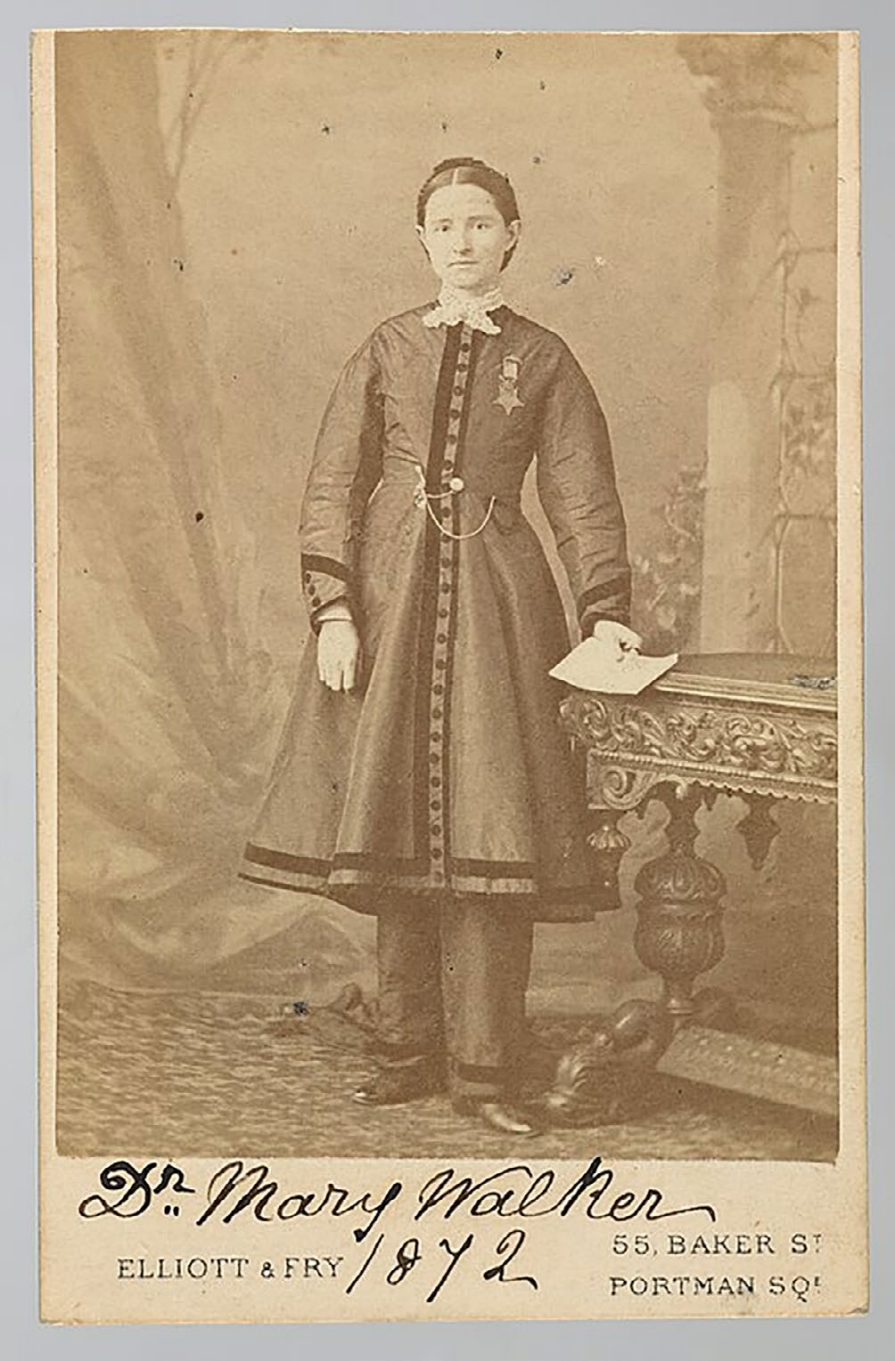
Mary Edwards Walker (1832–1919). First female U.S. Army surgeon. National Portrait Gallery, Smithsonian Institution, Washington.

Dr. Mary Edwards Walker (1832–1919), (Figure 1) graduated from the same medical school in 1855. She volunteered for the Union Army as a surgeon during the outbreak of the Civil War in 1861 but was rejected. Working as a nurse, her surgical skills in treating wounded soldiers soon brought her wide acclaim and led to her appointment as a Field Surgeon in the U.S. Army in 1964, the first female to achieve this position. After the war concluded in 1865, she was awarded the Congressional Medal of Honor for her Meritorious Service as an army surgeon and remains the only woman to receive this military decoration. Walker who famously wore pants and advocated for ‘dress reform’ was a strong advocate for women's rights. In 1916, her Medal of Honor was rescinded as she was regarded a civilian at the time of her valour. However, this did not stop her from wearing her medal during the remainder of her life. In 1977 President Carter legally restored the Medal of Honor to her name. Walker was inducted into the National Women's Hall of Fame in 2000, and her hometown unveiled a bronze statue in her honour in 2012 [20].

Dr. Elizabeth Garrett (1836–1917) later known as Elizabeth Garrett Anderson, became the first woman to qualify as a medical practitioner in the United Kingdom and the second to be registered on the British Medical Register [21]. Undaunted by the rejection of her application to several medical schools, she embarked on a course of private study, and instruction from accredited medical practitioners in 1860. Her enquiries into the possibility of being examined at Apothecaries' Hall caused consternation as the introduction of female medical practitioners had never been contemplated. According to the Apothecaries Act of 1815, the Society of Apothecary's examinations were open to all ‘persons’, provided they conformed to the regulations, and therefore in her favour. She passed the Society's Arts Examination in 1862, the first part of the professional exam in 1864 and the second part in 1865, thus gaining her medical Diploma (LSA). The loophole through which she gained entry was immediately closed to prevent other women including Sophia Jex‐Blake from following suit [22].

In the same year that Dr. Garrett Anderson qualified (1865), Dr. Wilhelmina Fergus, a graduate of the University of Pennsylvania, arrived as a ship's doctor in Australia and applied to the Victorian Medical Board for registration. Her application was refused by the Board and ridiculed in an editorial published in the Australian Medical Journal in 1865. This stated, ‘There is little fear in any British community that medical women will exist as a class. They will occasionally be imported like other curiosities, and the people will wonder at them just as it wonders at dancing dogs, fat boys and bearded ladies’. It would take another 25 years before Constance Stone, who was born in Tasmania and graduated from the University of Pennsylvania, became, in 1890, the first female doctor to be registered in Australia.

Garrett Anderson desire was to become a surgeon but like those women who came later, she was barred by certain social attitudes and educational requirements. Anatomy dissecting rooms were considered unhygienic and a danger to women's health. Exposure to blood and body fluids during surgery was thought to be too upsetting for the delicate female constitution. Women were deemed as too hysterical to be trusted in the operating theatre, and to lack the physical strength necessary to perform some operations. Overall, the occupation of surgery was regarded as too demanding, dangerous and vulgar for women with the result that respectable families were reluctant to allow their daughters to become surgeons [23]. The educational requirements to gain admission to the examinations of the various Royal Colleges of Surgeons stipulated specified courses, anatomy dissection, and a three‐year hospital apprenticeship or ‘residency’ with a surgeon, none of which were then open to women [23].

Dr. Garrett Anderson cofounded the London School of Medicine for Women (LSMW)—later known as the Royal Free Hospital School of Medicine—and was Dean of that School between 1883 and 1902. In 1895 she wrote to the Royal College of Surgeons of England requesting her female students be admitted to its examinations. This was rejected. In 1906 she delivered a petition signed by herself and 39 members of the staff of LSMW and the Royal Free Hospital, including male surgeons. There is little doubt that her active campaign played an important part in the eventual decision of the Royal College of Surgeons of England to admit women to its examinations in 1909. A summary of ‘the fight for the admission of women to the examinations of the College’ makes for interesting reading [24].

**FIGURE 2:**
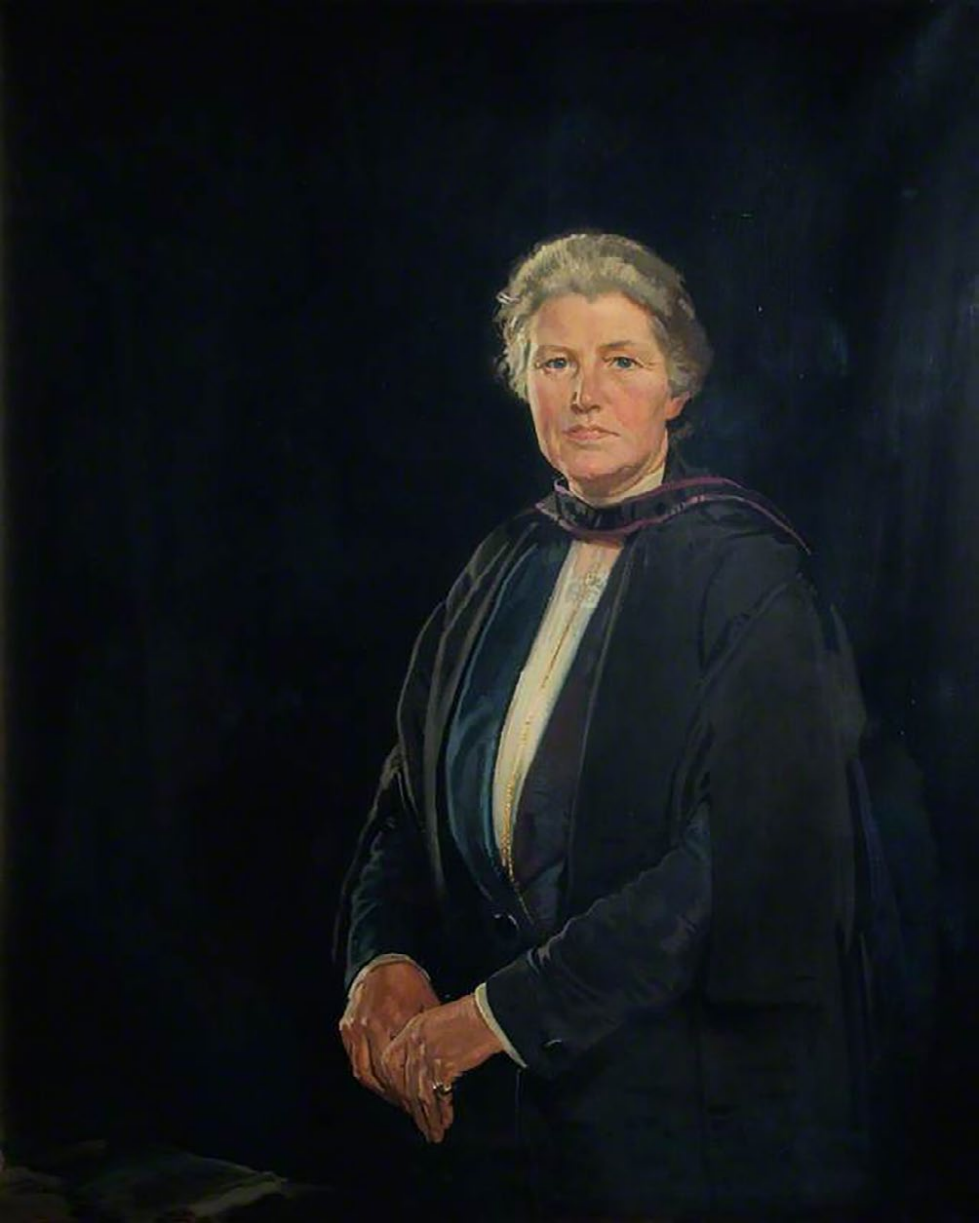
Louisa Aldrich‐Black. First woman to qualify as a surgeon in Britain. Portrait by William Orpen, University College London Hospitals.

The first woman to qualify as a surgeon in Britain was Dame Louisa Aldrich‐Blake (1865–1925) (Figure 2). Educated first at home, and later at Cheltenham Ladies' College where she excelled academically and in cricket and boxing. She entered the London School of Medicine for Women in 1887, and in 1892 passed the examination for the M.B. degree from the University of London with first‐class honours in medicine and obstetric medicine. The following year she graduated B.S. with first class honours and the gold medal in surgery. She obtained her M.D. in 1894 and her M.S. in in 1895, thus becoming the first woman in Britain to hold a higher degree in surgery [25]. She became the first woman to be appointed as a hospital surgical registrar, and the first female anaesthetist. Her M.S. enabled her to be appointed as the first female consultant surgeon in Britain. She undertook major colorectal and gynaecological surgery with great skill at the Royal Free Hospital and described a new surgical method for the treatment of rectal cancer in 1903 [26]. In 1914 she became Dean of the London School of Medicine for Women.

Aldrich‐Blake's work as a surgeon, educator, and her military efforts during WW1, received wide acclaim including Royal approval when she was created Dame Commander in the Order of the British Empire among the New Years Honours of 1925 [27]. Soon after her death in 1926, a memorial statue was erected in Tavistock Square Gardens in London, commemorating her many achievements. Her pioneering work changed British medical history and inspired a generation of young women to pursue a medical career including in surgery.

## 6 First Female Fellows of the Royal Colleges of Surgeons by Examination

**FIGURE 3:**
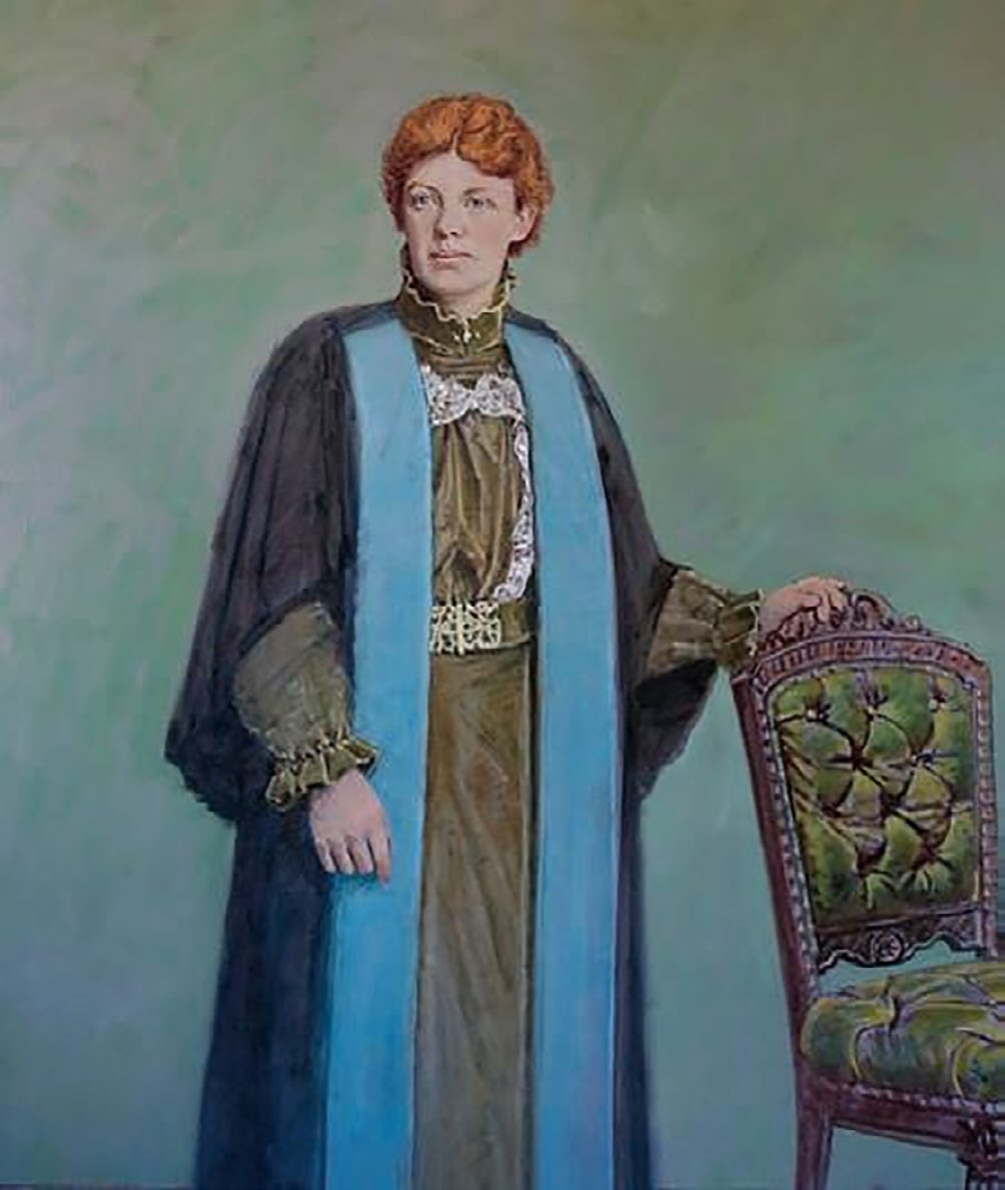
Emily Winifred Dickson. First woman to obtain the Fellowship of any Royal College by examination. Portrait by Mick O'Dea, Royal College of Surgeons in Ireland.

By the end of the nineteenth century, those who wished to practice as surgeons in the United Kingdom and Ireland, were expected to pass the Fellowship examination of one of the Royal Colleges of Surgeons. The first woman to do so was Dr. Emily Winifred Dickson (1866–1944) (Figure 3) who became a Fellow of the Royal College of Surgeons in Ireland (FRCSI) by examination in 1893. After being refused entry to study medicine at Trinity College Dublin, she enrolled at the Royal College of Surgeons in Ireland and obtained the Licentiate of the Royal Colleges of Physicians and Surgeons in 1891. In 1893 she graduated in medicine from the new Royal University of Ireland with first class honours. That same year she was awarded the FRCSI. In 1894 she was appointed as the first female gynaecologist at the Richmond Hospital in Dublin and in 1896 awarded her M.D. and M.A.O. both with honours. Further hospital appointments followed, and she was elected as Examiner in Midwifery and Gynaecology at the RCSI, a first for any woman in Ireland or Britain. Disappointment ensued when her application for the Chair in Obstetrics and Gynaecology at the RCSI was unsuccessful.

Dr. Dickson, who was married and ceased to practice medicine in 1899, went on to have five children. After her marriage effectively ended, she became the sole breadwinner for her family and moved to England where her children were in boarding schools. She held various medical posts, often taking short‐term medical appointments. Before her death in 1944, this brilliant woman noted in a letter, ‘the countless ways that I missed many priceless chances and opportunities’ through being unaware they existed [28]. On the 150th anniversary of Dickson's birth, the RCSI instituted the Emily Winifred Dickson Award to recognise women who have made an outstanding contribution in their field.

Dr. Eleanor Davies‐Colley (1866–1944) was the first woman to be awarded the Fellowship of the Royal College of Surgeons of England (FRCSEng) by examination. Born into an affluent and surgical family, she graduated from the London School of Medicine in 1907 and 3 years later was awarded her M.D. by the University of London. After obtaining her FRCSEng in 1911, she became a cofounder of the South London Hospital for Women and Children and later one of its surgeons. Otherwise known as ‘Adamless Eden’, the only male staff at this hospital were the engineer and the gardener! Davies‐Colley went on to work as an Obstetrician and Gynaecologist at the Elizabeth Garrett Anderson Hospital and as a surgeon at the Marie Curie Cancer Hospital. Recognised for her skilful and conscientious work, this altruistic, shy and humble person died unexpectedly from thyrotoxicosis at the age of 60 years. A lecture theatre was named in her memory at the Royal College of Surgeons of England [29].

The first woman to be awarded the Fellowship of the Royal College of Physicians and Surgeons of Glasgow (FRCS(G)) by examination was Dr. Jamini Sen (1871–1933). Born in Bengal and after graduating in Fine Arts, she enrolled at Calcutta Medical College where she was awarded a Licentiate in Medicine and Surgery in 1897. After a period as physician to the Royal Family in Nepal and head of Kathmandu Hospital, she was awarded a Duffin Fund scholarship. She travelled to Dublin to obtain a medical licence following which she went to Glasgow and passed the examination for FRCS (G) in 1912. Dr. Sen undertook further training in Berlin and London and then returned to India to join the Women's Medical Service. Working mainly in maternity in Countess of Dufferin funded hospitals, she later became head of Baldeodas Maternity Hospital in Kolkata in West Bengal. This remarkable woman broke many barriers facing female doctors in India and championed better access to medical care for female patients and children [30].

The Royal College of Surgeons of Edinburgh was the last of the surgical colleges in Great Britain and Ireland to admit women to the examination for its Fellowship (FRCSEd). After the Sex Disqualification (Removal) Act of 1919 was passed into law, it became illegal to exclude any woman from employment or to preclude her from joining the professions and professional bodies. Perhaps nudged by this Act, the College allowed its first female candidate Dr. Alice Headwards (1888–1973) to take its Fellowship examination [31]. Headwards (later Headwards Hunter) was born in India and educated in England. After obtaining a Licentiate of the Apothecaries Hall in Dublin in 1910, she returned to India where she gained further surgical experience in the Royal Army Medical Corps (RAMC) in Bombay. This was followed by a period as doctor‐in‐charge of Peshawar Municipal Hospital for Women and Children. She then went to Edinburgh where she sat and passed the Fellowship examination of the Royal College of Surgeons of Edinburgh (FRCSEd) in 1920, thus becoming the first woman to do so.

She returned to India where she made an enormous contribution particularly to the care of women and children. During the Second World War she assisted with the causalities and refugees on the Indian Burmese border and set up a convalescent home for British soldiers. At the request of the Bengal Government in 1943, she established a temporary hospital for child famine victims, caring for more than 400 at a time. For her services she was awarded the Kaiser‐i‐Hind Medal by the Indian Government in 1945. The Royal College of Surgeons of Edinburgh Hunter‐Doig Medal honours her achievements, as well as those of Dr. Caroline Doig, the first woman to be elected to the Council of that College.

Dr. Jessie Catherine Gray (1910–1978) was the first woman to be awarded the Fellowship in Surgery (FRCPSC) through examination, by the Royal College of Physicians and Surgeons of Canada. Born in Augusta, she moved to Toronto as a child and graduated with a B.Sc. from the University of Toronto in 1931. She obtained her M.D. with the gold medal for the highest academic standing in her class, the first woman to do so. Following internships at Toronto General Hospital, she enrolled on the prestigious Gallic Course in Surgery at the University of Toronto and graduated with a M.S. in 1939, again, the first woman to do so. In 1940 she became the first female surgical resident in Toronto, and the following year was awarded the FRCPSC.

Gray became the first woman to be registered as a surgeon in Canada although Dr. Jennie Smillie Robertson (1878–1919) was the first recorded Canadian surgeon. She became Chief of Surgery at the Women's College Hospital, a Fellow of the American College of Surgeons, and Assistant Professor at the University of Toronto. Known as the Canadian ‘First Lady of Surgery’, she was the first woman to be elected as a member of the Science Council of Canada in 1966 and was made a Life Fellow of the Academy of Medicine in 1968. In 1973 Dr. Gray received the Civic Award of Merit from the mayor of Toronto. An outstanding cancer surgeon, educator and researcher, Dr. Gray is regarded as a trailblazer and inspiration to generations of women surgeons and researchers [32].

**FIGURE 4:**
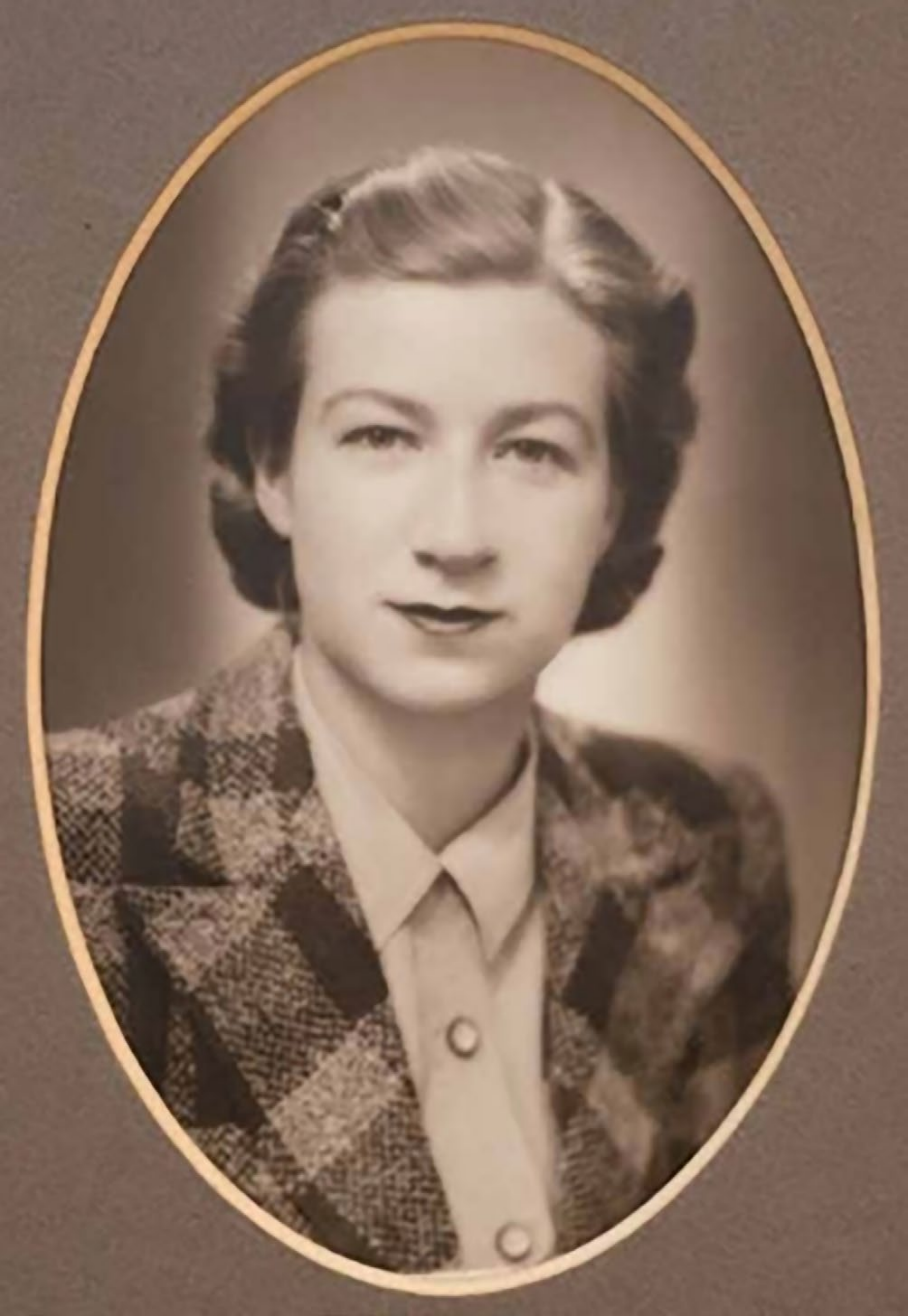
Lorna Verdun Sisley. First woman to obtain the Fellowship of the Royal Australasian College of Surgeons by examination. Picture in St Vincent's Hospital Melbourne Collection.

Although Dr. Lilian Violet Cooper became the first female Fellow of the Royal Australasian College of Surgeons (FRACS) at its foundation in 1927, this was based on her previous surgical experience. Dr. Lorna Verdun Sisely (1916–2004) (Figure 4) was the first woman to obtain the Fellowship by examination. Born in Wangaratta, Victoria, her desire to study medicine was at first refused by her father who held that ‘women should focus on getting married rather than on education’. Her response was to declare, ‘if I can't practice medicine, I will be no use for anything else’. After completing the first year of a science course at the University of Melbourne, she transferred to medicine and graduated in 1942 with first class honours and the Michael Ryan Scholarship in surgery. She became a junior resident at St Vincent's hospital during which her ability and potential were recognised by Sir Hugh Devine, who in turn encouraged her to embark on a surgical career. After gaining further surgical experience with the master surgeon Mr. Leo Doyle, she obtained the FRACS in 1947. In 1949 she gained her M.S.

That same year Dr. Sisely was awarded the Gordon Craig Travelling Scholarship which she used to further her studies in the UK and the U.S. She was appointed as a surgeon at the Queen Victoria Medical Centre and later became the founder and consultant surgeon of the Breast Clinic at the Monash Medical Centre. She also served as Dean of its Clinical School. She became a Fellow of the American College of Surgeon in 1962 and the first woman to be elected as a member of the Urological Society of Australasia in 1963. Recognised as a ‘fine surgeon with sharp diagnostic skills’, Sisley was appointed an Officer of The British Empire (OBE) in 1980 and awarded the Centenary Medal (CM) in 2001, both for her services to medicine. A champion of women in medicine, she always acknowledged the importance of the support, mentoring and sponsorship she received from Devine, Doyle and others [33].

## 7 First Woman To Be Board Certified by the American Board of Surgery

**FIGURE 5:**
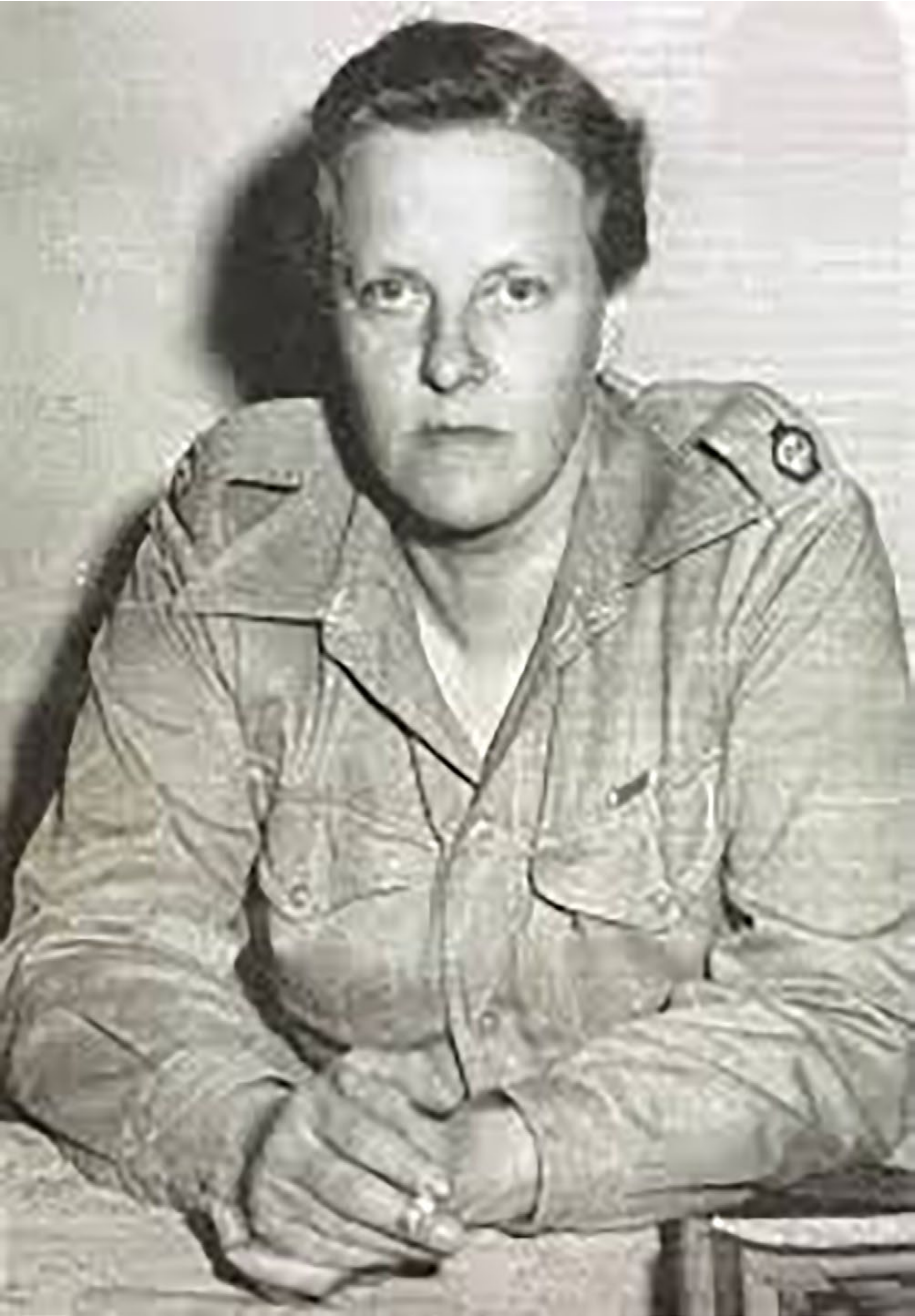
Barbara Bartlett Stimson. First woman surgeon to be certified by the Americal Board of Surgery. Photo taken in Rome 1945. Digital Collection of the National WW11 Museum, New Orleans.

Dr. Barbara Bartlett Stimson (1898–1986) (Figure 5) was the first female surgeon to be certified by the American Board of Surgery in 1940. After graduating with honours from Columbia University College of Physicians and Surgeons, Stimson completed her internship at the Columbia Presbyterian Medical Centre. She was then awarded a National Council Fellowship at Columbia University. Encouraged to pursue orthopaedic surgery by Professor William Darrach, she later commenced practice as an orthopaedic surgeon in 1934 and was admitted as a Fellow of the American College of Surgeons that same year.

Her application to serve as a medical officer in the American Army was rejected because she was a woman. Undaunted, she moved to the UK where she received a Commission in the British Army. Dr. Stimson went on to serve as an orthopaedic surgeon with the rank of Major throughout World War 11, completing tours in England, North Africa and Italy. She was awarded the Military Medal of a Member of the Order of the British Empire before returning to New York to continue practice. In 1950 she was awarded the Elizabeth Blackwell Medal. Dr. Stimson always held that proven skill and ability can be used to overcome unfounded biases, and her story helped to pave the way for future generations of female surgeons [34].

## 8 Key Themes From the Lives and Careers of These Female Surgeons

Analysis of the lives and careers of these women demonstrates several important themes. They established that women could excel as surgeons and medical researchers and play a vital role in the delivery of health care. Skill and determination were shown to overcome unfounded biases. Early recognition, career advice and mentoring increased the likelihood of their success. Lack of sponsorship and awareness of opportunities hindered some of their careers. These female surgeons were strong advocates for women's and children's health and gave a lifetime of service to the needy and neglected. Others helped to break down existing barriers against women doctors in different countries. Each of them became trailblazers and role models for subsequent generations of women and men.

Despite the success of these and of the countless female surgeons who have since followed them, a review of the current literature illustrates the continuing challenges faced by women who wish to train and practice as surgeons [35, 36, 37]. Although certain sociocultural beliefs and attitudes regarding gender roles still persist, interventions should ‘not unduly focus on gender, and address multiple factors’ [36]. Women surgeons tend to underestimate their competence and skills, are more prone to self‐doubt and imposter syndrome than men, and, if not promoted, may refrain from reapplying [38]. Introducing them by their first names instead of by their full names, professional titles and achievements is regarded as another bias. Women surgeons are considered to be ‘over‐mentored and under‐sponsored’ [39]. Continuing sponsorship and the inclusion of a male mentor is recommended [38]. Discrimination, bullying and sexual harassment of female surgical trainees and the ‘conspiracy of silence’ which often surrounds it remains prevalent in the workplace, a blot on the profession, and off‐putting to those women contemplating a career in surgery [40, 41].

For the satisfactory recruitment, education and training, career satisfaction and retention of women surgeons, the challenges and lifestyle issues which remain must be addressed. The educational organisations responsible for surgical education and training, and the healthcare leaders and hospital administrators as well as male surgical colleagues, must work together to foster the necessary changes. In addition, the structures that entrench inequality, and our own behaviour and attitudes, and those we experience every day must be faced up to [42]. Until these factors have been satisfactorily addressed, the professionalisation of surgery and of its practitioners the surgeons, which commenced in the mid‐eighteenth century will still not be complete by the centenary of the Royal Australasian College of Surgeons in 2027 [43].

## Disclosure

The author has nothing to report.

## Data Availability

This manuscript does not contain any data.
